# Arytenoid dislocation related to an uneventful endotracheal intubation: a case report

**DOI:** 10.1186/1757-1626-1-251

**Published:** 2008-10-20

**Authors:** Nimet Senoglu, Hafize Oksuz, Nadiye Ugur, Zafer Dogan, Ali Kahraman

**Affiliations:** 1 Kahramanmaras Sutcu Imam University Medical Faculty, Department of Anesthesiology and Reanimation, Kahramanmaras, Turkey; 2 Kahramanmaras Sutcu Imam University Medical Faculty, Department of Otolaryngology-Head and Neck Surgery, Kahramanmaras, Turkey

## Abstract

**Introduction:**

Invasive methods currently applied to the respiratory tract may result in impaired movement of the cricoarytenoid joint with hoarseness and immobility of the vocal ligament. Hoarseness after tracheal intubation is reported as a high incidence in patients who receive general anaesthesia. In most cases, the symptoms are temporary and improve within several days. We report this case for emphasizing that early diagnosis of arytenoid cartilage dislocation is important even in nontraumatic cases.

**Case presentation:**

We present the case of a 19-year-old Caucasian male who developed arytenoid cartilage dislocation associated with uneventful endotracheal intubation and anesthesia.

**Conclusion:**

Arytenoid subluxation should be considered whenever any of the symptoms mentioned occur following endolaryngeal manipulation, and they become persistent, as recovery becomes difficult if appropriate treatment is not started immediately.

## Introduction

Endotracheal intubation, laryngoscopy, bronchoscopy, and other invasive methods, which are often applied to the respiratory tract, may result in injuries of the airway. These complications include submucosal hemorrhage, granuloma formation, subglottic edema or laryngitis, impaired movement of one or both vocal folds caused by recurrent laryngeal nerve damage, and cricoarytenoid joint dysfunction, and may be characterized by hoarseness or stridor [1,2].

Hoarseness is a common postoperative complication and reported as an incidence between 14.4% and 50% of patients. Prolonged or permanent hoarseness may occur in 1% of patients [1].

Due to the non-sensitive symptomatology, diagnosis of the cricoarytenoid injury can be delayed, and recovery becomes difficult if appropriate treatment is not started early. The symptoms are temporary and improve within several days in most cases. In the case of persistent hoarseness, specialists should consider arytenoid cartilage dislocation. Fortunately this is a rare event, the frequency related to direct laryngoscopy has been reported to be 0.023% [3].

In this report, we present a case of arytenoid cartilage dislocation caused by an apparently straightforward tracheal intubation and anesthesia.

## Case presentation

**Figure 1:**
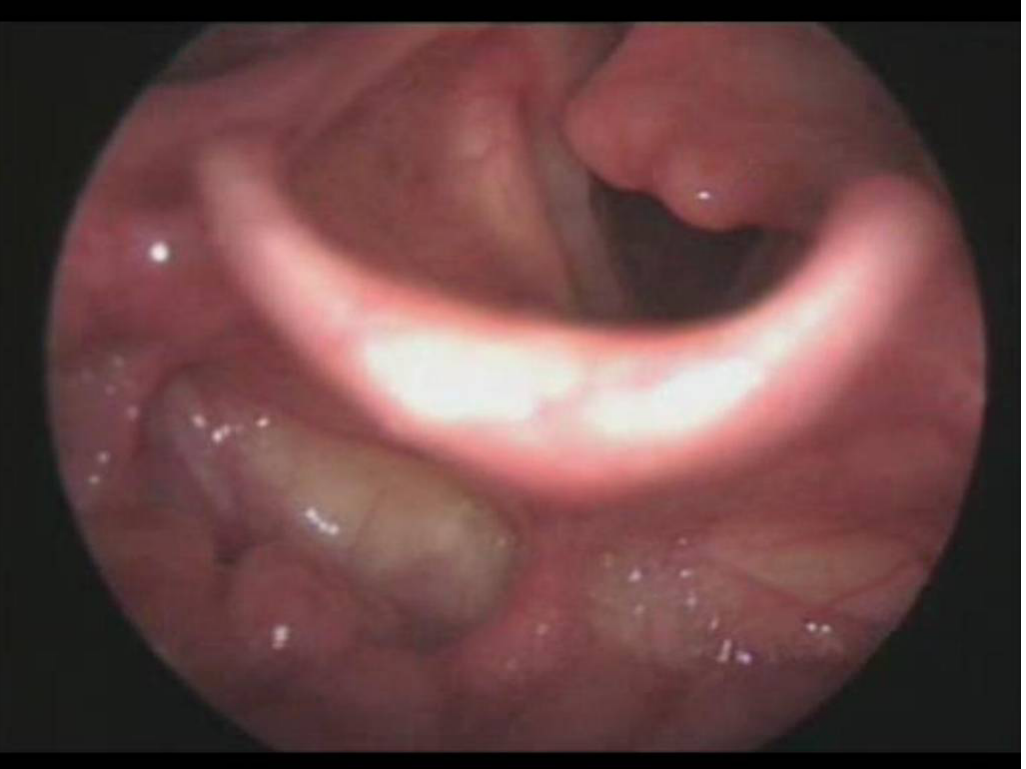
On the 6^th ^day after the operation, fiberoptic laryngoscopy was seen as anteromedial dislocation of the left arytenoid cartilage.

A 19-yr-old Caucasian male was scheduled for a septorhinoplasty operation. He had no history of laryngeal disorders and there was no apparent diabetes mellitus, chronic renal failure, chronic corticosteroid use, laryngeal malacia, acromegaly or other factors that might weaken the cricoarytenoid joint. His past medical history, physical examination and laboratory examination were unremarkable (preoperative physical status clasification of patients according to the American Society of Anesthesiologists; ASA I). Physical examination revealed a class 2 airway. Mallampati Intubation Score and other airway measurements were normal. After securing intravenous access and beginning standard monitoring, anesthesia was induced with thiopental (8 mg.kg^-1^), rocuronium (0.6 mg.kg^-1^), and remifentanyl (0.5 mcg.kg^-1^) intravenously. Complete muscle relaxation was provided before insertion of the tube. At the visualisation of larynx with direct laringoscopy (Macintosh 4 laryngoscope blade) the epiglottis was short but the glottis was Grade II (Cormack-Lahene). On the first attempt, a 8.0-mm endotracheal tube was smoothly inserted into the trachea without difficulty and fixed at the middle. The patient did not cough during the intubation. During the four hour operative procedure, anesthesia was maintained with nitrous oxide, oxygen, isoflurane, remifentanyl (0.25 mcg.kg^-1^.min^-1^) and rocuronium. The surgery was uneventful. After the operation, the endotracheal tube cuff was deflated and removed without any difficulty, severe coughing and vomiting did not occur at this period. On the first day, the patient complained of moderate hoarseness. He was discharged on the second day and still complained of persistant hoarseness on the 6^th ^day. He had no symptoms other than hoarseness. On the 6^th ^day after the operation, fiberoptic laryngoscopy was performed by an otorhinolaryngologist and revealed anteromedial dislocation of the left arytenoid cartilage (figure 1). Surgical reduction, under the general anesthesia, was scheduled on the 9^th ^post-operative day. The dislocation of the arytenoid cartilage was visualised under rigid broncoscopy and posterolateral reposition by spatula and steroid injection were performed. The hoarseness was relieved gradually in 6 weeks. Written informed consent was obtained from the patient for publication of this case report and accompanying images.

## Discussion

Injuries of the larynx are a well known complication of anesthesia and range from moderate to severe. Incidence of hoarseness is common, varying between 14.4 and 50%. Most cases are temporary and improve within several days [2,4]. If hoarseness persists it could indicate severe injury, including impaired movement of one or both vocal folds caused by recurrent laryngeal nerve damage, and/or cricoarytenoid joint dysfunction. Endotracheal tube size, cuff design, and cuff pressure, as well as demographic factors such as sex or even the type of surgery are reported as risk factors [2].

Research has been done to investigate the mechanism of arytenoid dislocation. The preferred theories are incomplete neuromuscular blockage, motor reactions during endotracheal intubation, or direct trauma to the cricoarytenoid joints leading to joint cavity hemorrhage or serosynovitis. After this, pathological process can be followed by adhesion of articular surfaces or periarticular structures that fix the arytenoid in an abnormal position. Delay in diagnosis and treatment can consequently lead vocal fold immobility to occur. Typical subluxation leads to the arytenoid cartilage shifting in an anterior-medial or a posterior-lateral direction [2]. In this case, a stylet was not used during insertion of the tube, no gastric catheter was inserted and the patient did not cough during intubation.

The primary symptom is persistent hoarseness in adults and respiratory compromise in pediatric and neonatal cases [5]. Some symptoms such as dysphagia, sore throat and stridor may be caused by arytenoid cartilage dislocation. Some investigators reported that anterior subluxations of the arytenoid may be better tolerated with persistent hoarseness while posterior subluxations are associated with severe sore throat and odynophagia, as well as hoarseness [6]. In the presenting case the primary symptom was hoarsenes and the patient had no other symptoms.

Reported occurrences of arytenoid cartilage subluxation are rare. Several risk factors leading to laryngeal injury have been identified in the literature [4]. It has been reported that the use of lighted stylet, laryngeal mask airway and McCoy laryngoscope, endotracheal intubation with double lumen tube and cases of difficult intubation are associated with arytenoid cartilage dislocation [3]. Chronic disease such as laryngomalacia, renal insufficiency, acromegaly, or chronic glucocorticoid intake have also been reported to be predisposing factors [2,7].

In our patient, the cause of arytenoid cartilage dislocation was unclear, the intubation was not traumatic. In the literature, a case has reported as a complications of the uneventful and apparently straightforward endotracheal intubation and anesthesia [8]. Mencke and collagues examined influence of intubation conditions and intraoperative factors on pH and vocal cord sequelae [4]. They reported that excellent intubating scores were less frequently associated with either pH or vocal cord sequelae. Increasing the duration of surgery led to mucosal damage caused by the endotracheal tube and increased incidence of pH. Independently of the intubation conditions, laryngeal damage can be result from surgery of the larynx and surgery of the nose may postoperatively lead to temporary changes in the voice, pH [4]. These factors may explain why laryngeal damage could be observed in our patient.

## Conclusion

Recovery from arytenoid cartilage dislocation is difficult if appropriate treatment is delayed [3]. Early treatment is essential to avoid possible complications caused by the loss of normal sphincteric function of the larynx. Although arytenoid cartilage dislocation following uneventful intubation and anesthesia is a rare event, it is important that the anesthesiologist should suspect injury to the vocal folds or cricoarytenoid joints in case of persistent hoarseness. The specialist should accomplish arytenoid cartilage relocation as soon as possible after recognition of arytenoid subluxation as allowed by patient status.

## Competing interests

The authors declare that they have no competing interests.

## Authors' contributions

NS participated in the patient's management, drafted the manuscript. HO and NU performed the case management. ZD participated in drafting of manuscript. AK participated in the patient's diagnosis and management. All authors read and approved the final manuscript.

## Consent

Written informed consent was obtained from the patient's for publication of this case report and accompanying images. A copy of the written consent is available for review by the Editor-in-Chief of this journal.
